# Supplementary material for: Impact-induced changes in source depth and volume of magmatism on Mercury and their observational signatures
Source: Nat Commun. 2017 Dec 5;8:1945. doi: 10.1038/s41467-017-01692-0 (PMC5717040; doi:10.1038/s41467-017-01692-0)
Supplement: Supplementary file 1 — Supplementary Information [file 41467_2017_1692_MOESM1_ESM.pdf]

Supplementary Table 1: Bulk abundances of heat-producing elements potassium (K), thorium (Th), and uranium (U), for three compositional models and corresponding heat production in the mantle today, H_0 and at 4.5 Ga, $H_{4.5}$. The enrichment factor used for each model is indicated with Λ. The values of H are obtained with a mass balance that takes into account the observed surficial abundances and the thickness of the crust[1]. The enrichment factor is based on the ratio between the amount of K on the surface (1288 ppm, Ref. 2) and in the model.

Model	U (ppb)	Th (ppb)	K (ppm)	H_0 (pW/kg)	$H_{4.5}$ (pW/kg)	Λ
Enstatite Chondrite[3] (EH-HPE)	9	30	800	4.42	39.38	1.61
Carbonaceous Chondrite[4] (CI-HPE)	7	29	550	4.70	24.02	2.34
Bulk Silicate Earth[5] (BSE-HPE)	17	63	190	1.96	5.71	6.78

Supplementary Table 2: List of the parameters used in the simulations. Where needed, superscripts in the "Description" column point to the studies from which the values have been taken.

Symbol	Description	Value	Units
R_{P}	Planetary radius [6]	2439	km
R_{CMB}	Core radius [7]	2020	km
g	Surface gravity [8]	3.7	m/s^2
T_{S}	Surface temperature	440	K
T_{CMB}	Initial CMB temperature	1900	K
ρ	Mantle density [7]	3380	kg/m^3
ρ_{c}	Core density [7]	6980	kg/m^3
C_{P}	Mantle heat capacity [9]	1200	J/kg/K
$C_{\mathrm{P,c}}$	Core heat capacity [9]	850	J/kg/K
L_{m}	Latent heat of melting [10]	4.19×10^5	J/kg
α	Mantle thermal expansivity [10]	3×10^{-5}	K^{-1}
α_{c}	Core thermal expansivity [9]	5.8×10^{-5}	K^{-1}
k	Mantle thermal conductivity [11]	4	W/m/K
k_{Reg}	Megaregolith thermal conductivity [11]	0.2	W/m/K
d_{Reg}	Megaregolith thickness [11]	0 or 5	km
ν_{Ref}	Reference viscosity	$10^{19} - 10^{21}$	Pa s
E	Activation energy [12]	3.03×10^5	J/mol
V	Activation volume [12]	1×10^{-5}	m^3/mol
T_{Ref}	Reference temperature	1600	K
P_{Ref}	Reference pressure	3	GPa

Supplementary Table 3: Solidus and liquidus curves used in the simulations. Temperature T is in Kelvin and pressure P in GPa. Coefficients for the melting curves of Katz et al.[13] are taken from their Table 2. The solidus of Namur et al.[14] is from the caption of their Fig. 6, the liquidus has been obtained by a fit to the liquidus curve of their Fig. 6. The curves are plotted in Supplementary Fig. 1

Model ($T = A + BP + CP^2$)	A [K]	B [K/GPa]	C [K/GPa^2]
Solidus of Katz et al.[13]	1358.85	132.9	-5.1
Liquidus of Katz et al.[13]	2053.15	45.0	-2.0
Solidus of Namur et al.[14]	1421.15	177.0	-12.2
Liquidus of Namur et al.[14]	1933.28	64.5	-0.048

Supplementary Table 4: List of the parameters used in the comparison between the 1D and the 2D simulations. Parameters for the 2D simulation are taken from Supplementary Table 2, except for the megaregolith thickness, reference viscosity, and HPE model, whose values are listed in the upper part of the table. The bottom part of the table lists additional parameters required in the 1D model[11] that have no correspondence in the 2D model.

Symbol	Description	Value	Units
Parameters common to 1D and 2D simulations			
$d_{\rm Reg}$	Megaregolith thickness	5	km
$\nu_{\rm Ref}$	Reference viscosity	10^{21}	Pa s
Λ	Crustal enrichment factor	2.7	
U	Initial uranium concentration	$90/\Lambda$	ppb
Th	Initial thorium concentration	$155/\Lambda$	ppb
K	Initial potassium concentration	$1288/\Lambda$	ppb
Additional parameters for 1D simulations			
$T_{\rm m,0}$	Initial mantle temperature	1608	K
dT	Initial Core-Mantle Temperature difference	292	K
$D_{\rm c,0}$	Initial crustal thickness	5	km
$D_{\rm l,0}$	Initial lithospheric thickness	max(150, $D_{\rm c,0}$)	km
$dT_{\rm sol}$	Solidus increase due to melt	150	K
$f_{\rm c}$	Fraction of extractable crust	0.4	
dV/V	Volume change upon silicate melting	0.03	
$\rho_{\rm dep}$	Depleted mantle density	$\rho + 50$	kg/m^3
$C_{\rm P,magma}$	Magma heat capacity	1000	J/kg/K
$\rho_{\rm c}$	Crustal density	2900	kg/m^3

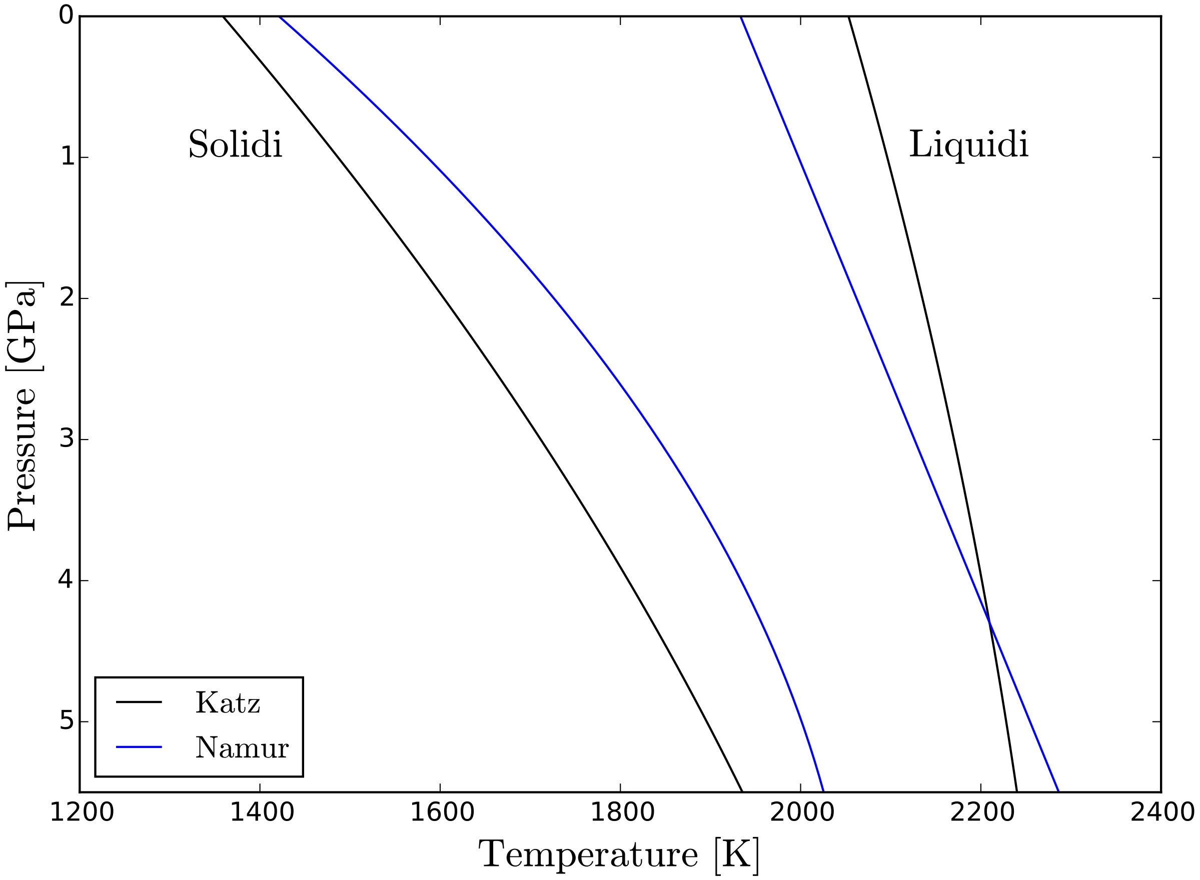

Supplementary Figure 1: **Melting curves.** Liquidus and solidus curves used in the simulations[13,14].

The curves are plotted with the parameterizations of Supplementary Table 3.

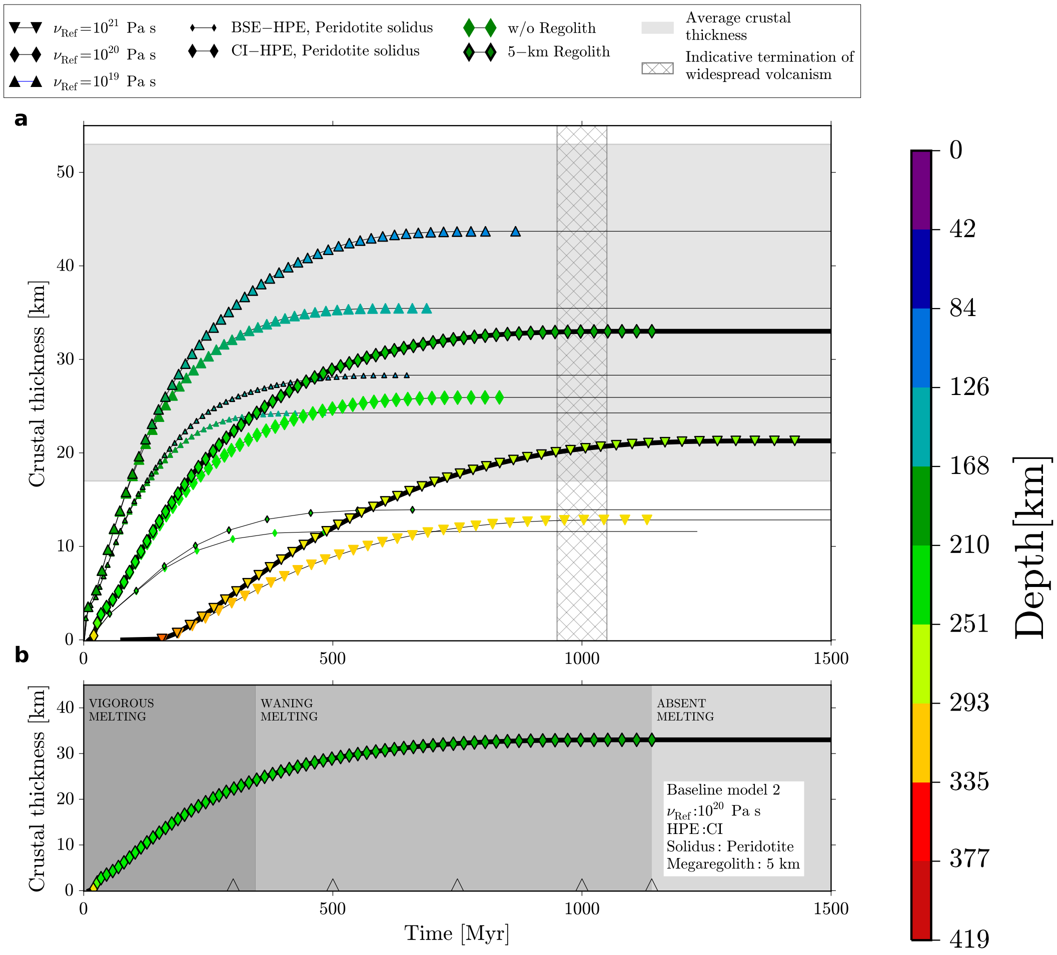

Supplementary Figure 2: **Crustal thickness from decompression (i.e., convective) melting as a function of time.** Same as Fig. 1 of the main text, but for the CI-HPE models (same set plotted in Fig. 1a) and the BSE-HPE (see Supplementary Table 1 for abundances). **(a)** Each set of data corresponds to different values of the reference viscosity (symbol shape), initial amount of heat-producing elements in the mantle (HPE, symbol size), and presence of a surficial megaregolith layer (indicated by a black contour). All models use a dry peridotite solidus[13]. Symbol colour indicates the average depth of the melt source region, according to the colourbar. For each model, the rightmost symbol plotted corresponds to the termination of convective-melt production. (Continues on the next page.)

Supplementary Figure 2: (Cont.) Models consistent both with the inferred crustal thickness[1] (grey horizontal band) and the duration of volcanic activity[15] (hashed vertical band) are drawn with a thicker black line. No BSE-HPE model is compatible with the constraints. **(b)** The three mantle melting regimes are shown for the model indicated in the white box, which, however, does not fit the lid thickness constraint (Fig. 2). The triangles along the time-axis indicate the times of impact for the simulations shown in Supplementary Fig. 5.

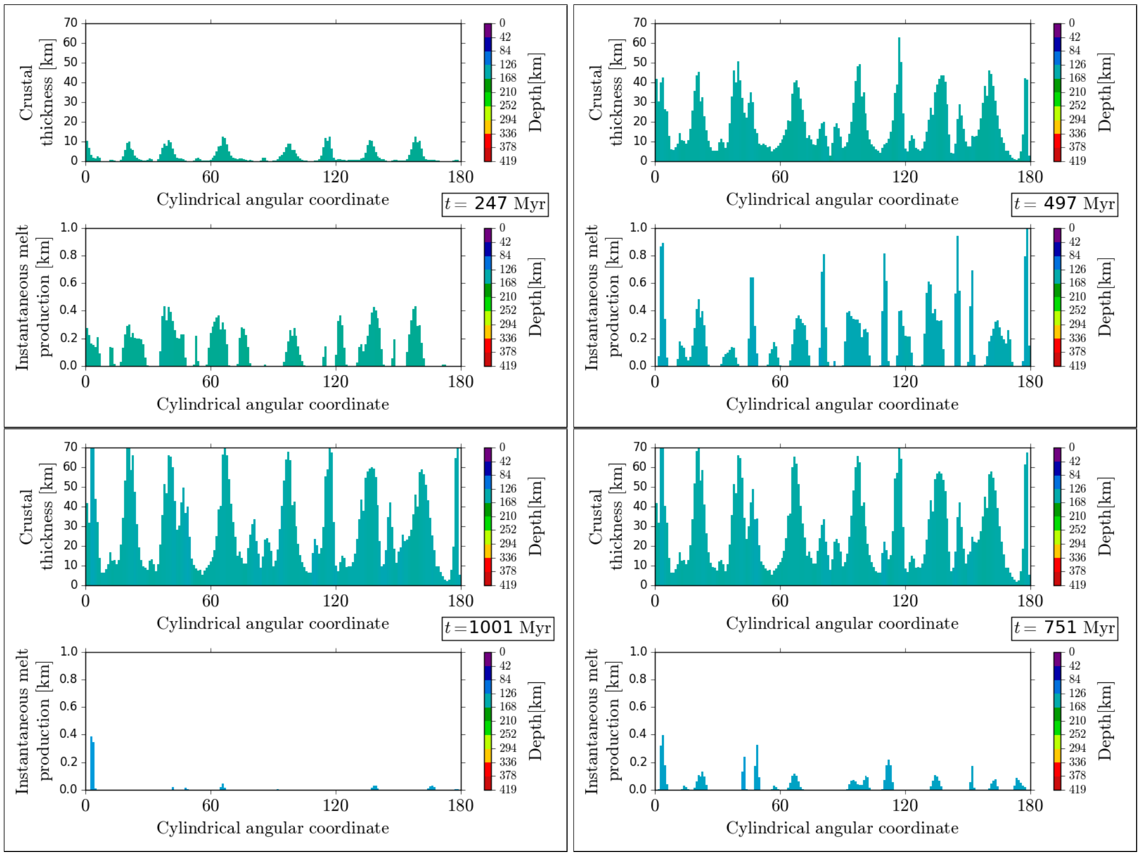

Supplementary Figure 3: **Melt production from convection.** In each frame the top histogram shows the cumulative melt produced (in terms of an equivalent crustal thickness) as a function of the cylindrical angular coordinate. The bottom histogram shows the instantaneous melt produced in terms of an equivalent thickness. The bars are colour-coded according to the characteristic depth of the melt. These data are for our baseline model (Fig. 1b). From top left clockwise the frames refer to 247, 497, 751, and 1001 Myr, respectively. The source depth is relatively constant throughout the entire evolution (see also Fig. 2 and Supplementary Fig. 4). Melt production is mostly associated with upwellings, and this explains the "spiky" appearance of the histograms.

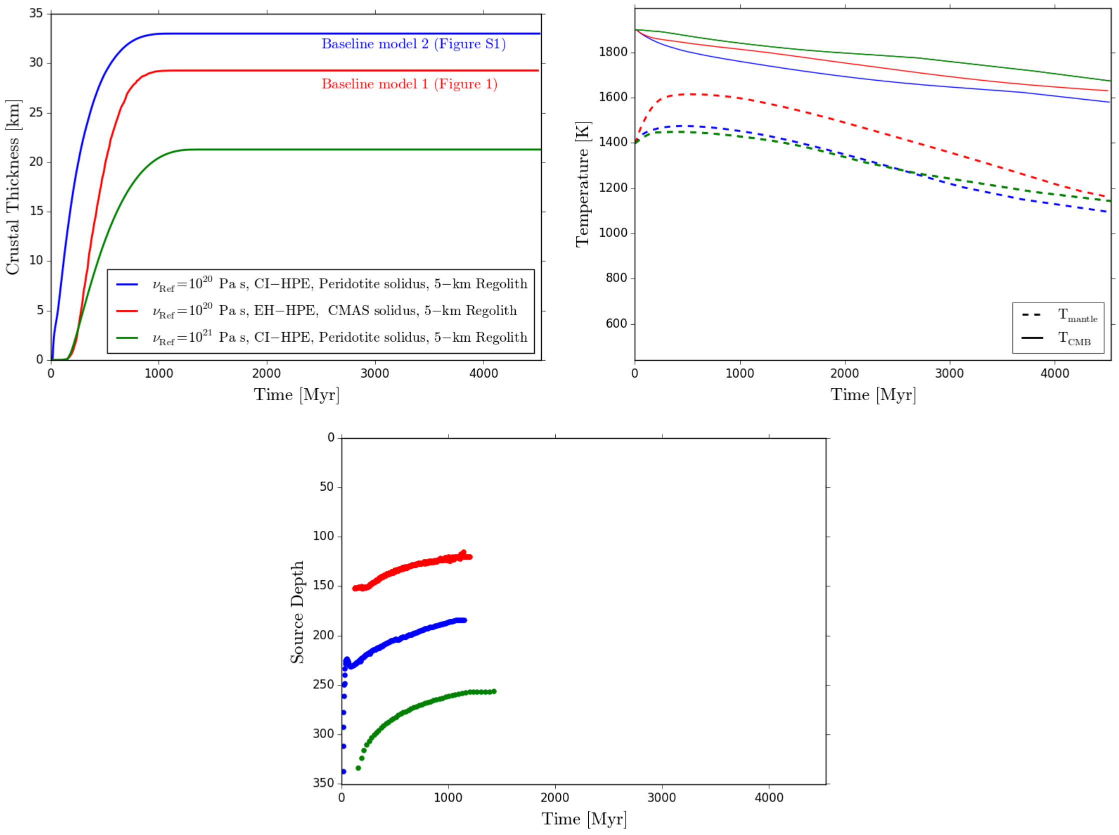

Supplementary Figure 4: **Models compatible with the volume of the crust and timing of major volcanic eruptions (thick black lines in Fig. 1a). Top left)** Evolution of the crustal thickness. **Top right)** Evolution of the core–mantle boundary (CMB) temperature and of the average mantle temperature. **Bottom)** Evolution of the depth of the source region for convective melt.

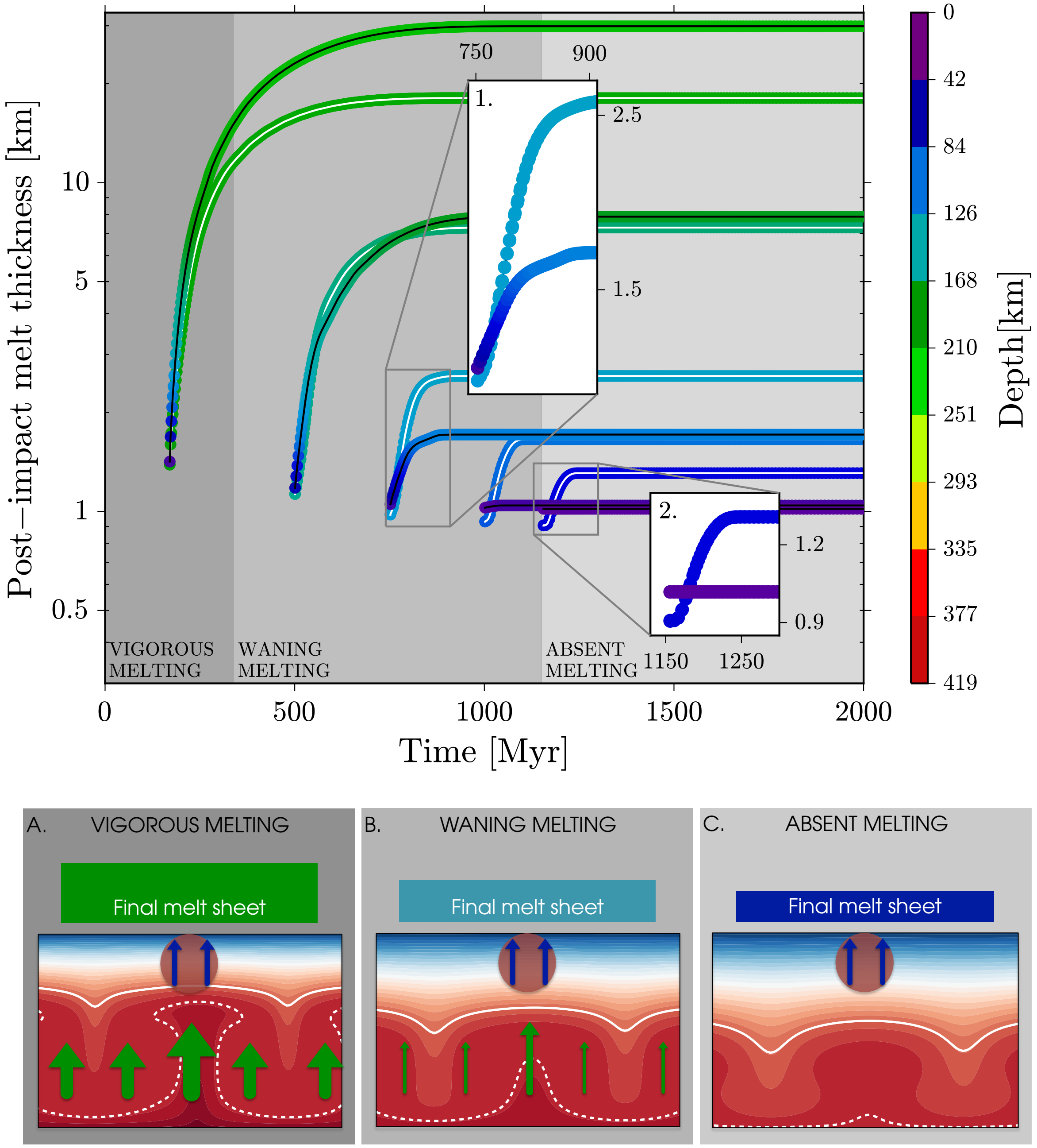

Supplementary Figure 5: **Melt production following an impact forming a Caloris-sized basin. Top)** Same as Fig. 5, but using the baseline model 2 (Supplementary Fig. 2b) and for the impact occurring at 170, 500, 750, 1000, and 1150 Myr. The impactor has a diameter of 92 km and hits the target with a velocity of 42.5 km/s and an impact angle of 45°. For each epoch the cases for the impact occurring over an upwelling and a downwelling are plotted. The impact over the downwelling (black line) initially extracts shallower material with respect to the impact over the upwelling (white line). (Continues on the next page.)

Supplementary Figure 5: (Cont.) The grey background indicates the melting regime as in Supplementary Fig. 2b, The insets zoom on the first few tens of Myr after the impact events at 750 and 1150 Myr. For impacts happening in the vigorous melting regime and early in the waning melting regime, the depth of the source region for the post-impact melt is rapidly controlled by the contribution of the convective melting (Cartoons A and B below). For the impact at 1150 Myr the contribution of convection melting is almost absent, and the melt is the result of partial melting in the shallow mantle (Cartoon C). The post-impact melt thickness represents an upper bound for the melt sheet thickness within the basin. **Bottom)** For the three melting regimes, the cartoons illustrate the temperature field in the mantle (background red/blue field), the impact-induced thermal anomaly (spherical red shape), and the melting contribution both from convection (green arrows) and from post-impact melting (blue arrows). The white lines represent a cold (solid) and a hot isotherm (dashed). The area of the arrows qualitatively indicates the amount of melt produced. More melt production is associated with upwellings (Supplementary Fig. 3). The thickness and source depth of the final melt sheet depends on the relative contribution of the two sources of melt, and is represented by the boxes labeled "Final melt sheet".

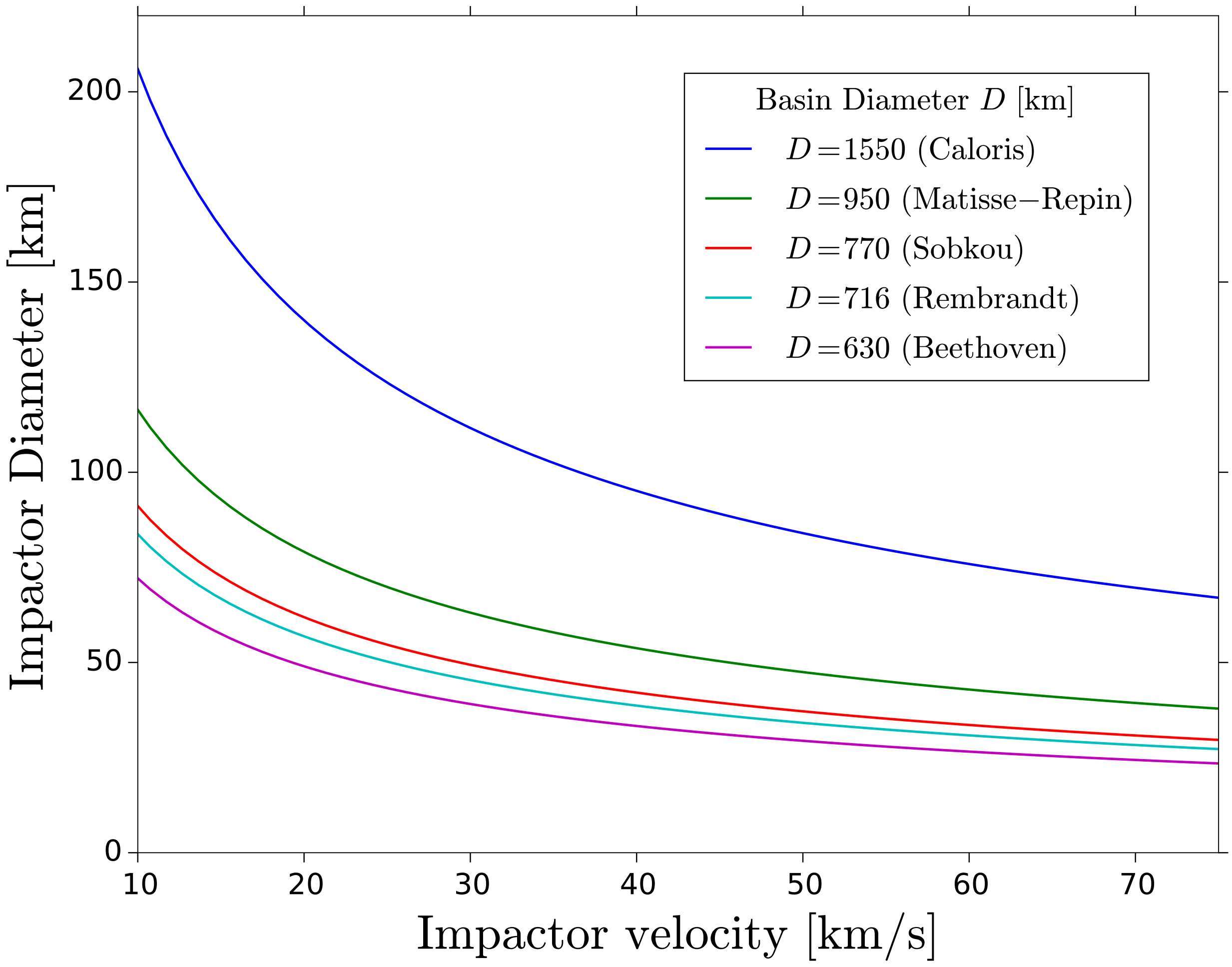

Supplementary Figure 6: **Impactor diameter and velocity.** Relation between impactor's diameter and encounter velocity required to form basins of different sizes on Mercury. Each curve refers to a particular basin, whose name and diameter are indicated in the legend. The curves are calculated assuming an impact angle of $45°$.

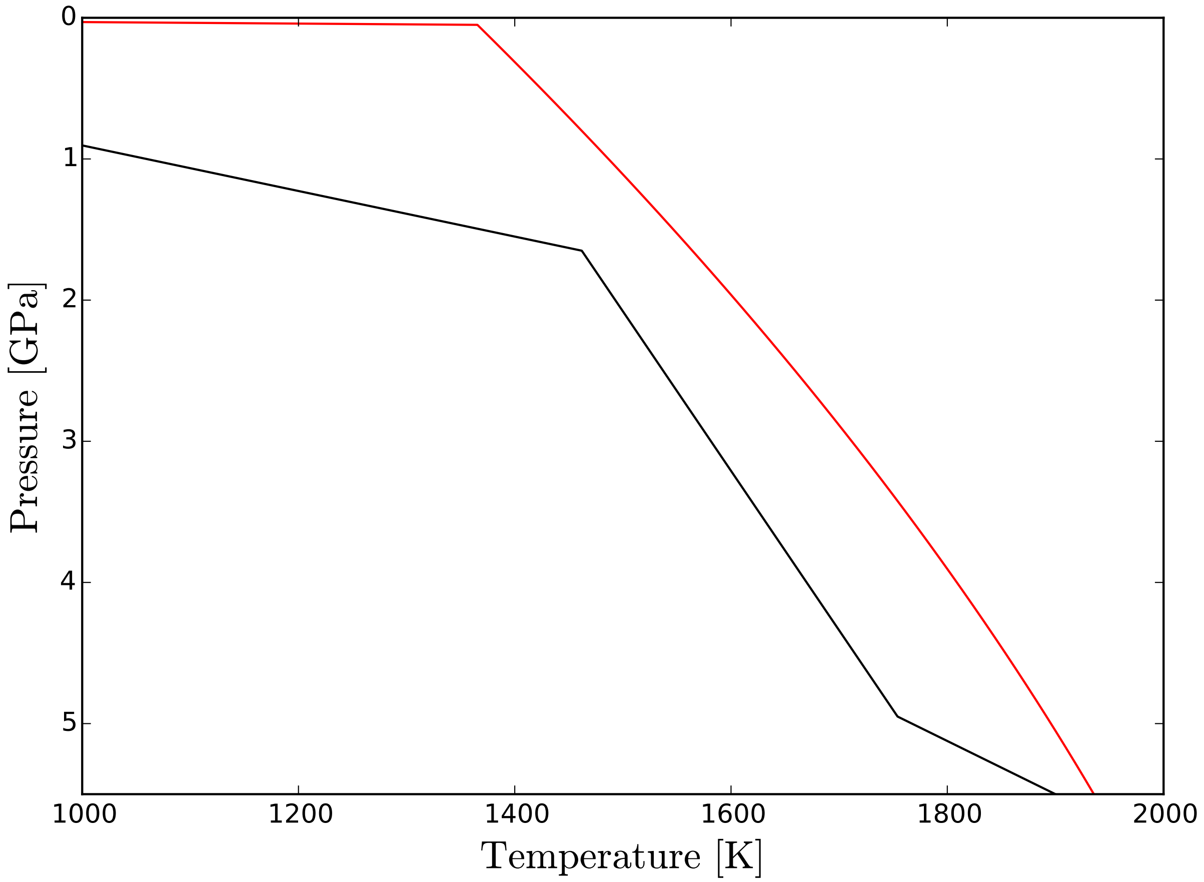

Supplementary Figure 7: **Initial temperature profile.** Initial temperature profile for the all the models presented in the paper (black) except for the hot models of Supplementary Fig. 8 (red). The surface temperature, corresponding to 0 GPa, is in both cases set at 440 K.

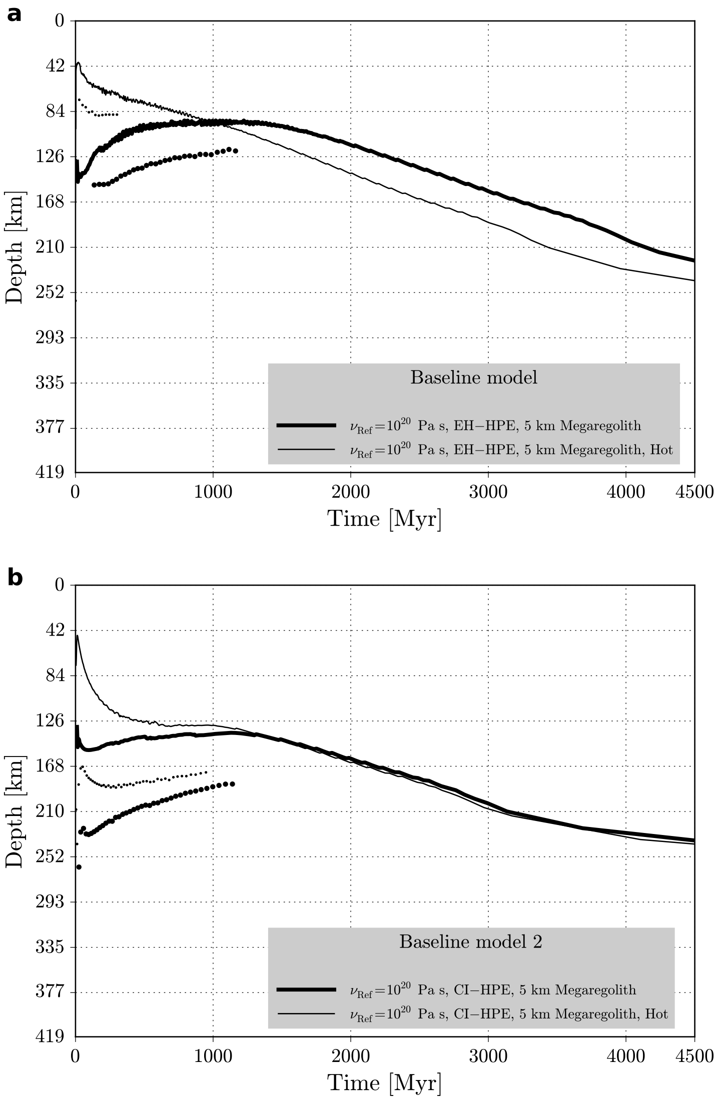

Supplementary Figure 8: **Lid thickness evolution.** Temporal evolution of the stagnant lid thickness (solid lines) and of the source depth of convective melts (dots). In each panel the two cases plotted correspond to a baseline model (thick line), and to the same model initialized with a temperature profile at the solidus (thin line). **(a)** Baseline model (Fig. 1b). **(b)** Baseline model 2 (Supplementary Fig. 2b).

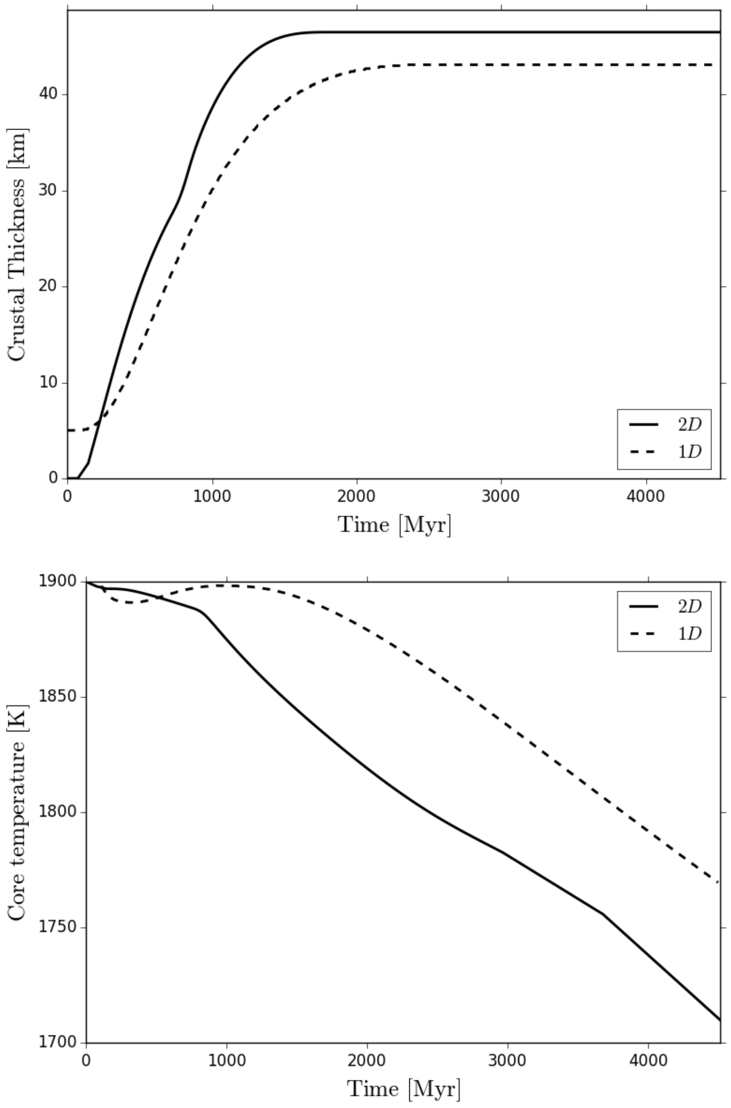

Supplementary Figure 9: **Comparison of 2D thermal evolution models with 1D parameterized models.** Comparison of crustal production (top) and temperature of the core (bottom) between a 1D parameterized evolution model[11] (dashed lines) and our thermal evolution model (solid lines). The parameterized model assumes a 5-km-thick primordial crust.

Supplementary References

1. Padovan, S., Wieczorek, M. A., Margot, J.-L., Tosi, N., and Solomon, S. C. Thickness of the crust of Mercury from geoid-to-topography ratios. *Geophys. Res. Lett.*, 42(4):1029–1038, 2015. doi: 10.1002/2014GL062487.

2. Peplowski, P. N., Lawrence, D. J., Rhodes, E. A., Sprague, A. L., McCoy, T. J., Denevi, B. W., Evans, L. G., Head, J. W., Nittler, L. R., Solomon, S. C., Stockstill-Cahill, K. R., and Weider, S. Z. Variations in the abundances of potassium and thorium on the surface of Mercury: Results from the MESSENGER Gamma-Ray Spectrometer. *J. Geophys. Res.*, 117:E00L04, 2012. doi: 10.1029/2012JE004141.

3. Wasson, J. T. and Kallemeyn, G. W. Compositions of chondrites. *Phil. Trans. R. Soc. London A*, 325:535–544, 1988. doi: 10.1098/rsta.1988.0066.

4. McDonough, W. F. and Sun, S. S. The composition of the earth. *Chem. Geol.*, 120:223 – 253, 1995. doi: http://dx.doi.org/10.1016/0009-2541(94)00140-4.

5. Lyubetskaya, T. and Korenaga, J. Chemical composition of Earth's primitive mantle and its variance: 1. Method and results. *J. Geophys. Res. Solid Earth*, 112:B03211, 2007. doi: 10.1029/2005JB004223.

6. Perry, M. E., Neumann, G. A., Phillips, R. J., Barnouin, O. S., Ernst, C. M., Kahan, D. S., Solomon, S. C., Zuber, M. T., Smith, D. E., Hauck, S. A., Peale, S. J., Margot, J.-L., Mazarico,

E., Johnson, C. L., Gaskell, R. W., Roberts, J. H., McNutt, R. L., and Oberst, J. The low-degree shape of Mercury. *Geophys. Res. Lett.*, 42:6951–6958, 2015. doi: 10.1002/2015GL065101.

7. Hauck, S. A., II, Margot, J.-L., Solomon, S. C., Phillips, R. J., Johnson, C. L., Lemoine, F. G., Mazarico, E., McCoy, T. J., Padovan, S., Peale, S. J., Perry, M. E., Smith, D. E., and Zuber, M. T. The curious case of Mercury's internal structure. *J. Geophys. Res. Planets*, 118: 1204–1220, 2013. doi: 10.1002/jgre.20091.

8. Mazarico, E., Genova, A., Goossens, S. J., Lemoine, F. G., Neumann, G. A., Zuber, M. T., Smith, D. E., and Solomon, S. C. The gravity field, orientation, and ephemeris of Mercury from MESSENGER observations after three years in orbit. *J. Geophys. Res. Planets*, 119: 2417–2436, december 2014. doi: 10.1002/2014JE004675. in press.

9. Tosi, N., Grott, M., Plesa, A.-C., and Breuer, D. Thermochemical evolution of Mercury's interior. *J. Geophys. Res. Planets*, 118:2474–2487, 2013. doi: 10.1002/jgre.20168.

10. Roberts, J. H. and Barnouin, O. S. The effect of the Caloris impact on the mantle dynamics and volcanism of Mercury. *J. Geophys. Res. Planets*, 117:E02007, 2012. doi: 10.1029/2011JE003876.

11. Grott, M., Breuer, D., and Laneuville, M. Thermo-chemical evolution and global contraction of mercury. *Earth Planet. Sc. Lett.*, 307:135–146, 2011. doi: 10.1016/j.epsl.2011.04.040.

12. Jackson, I., Faul, U. H., Suetsugu, D., Bina, C., Inoue, T., and Jellinek, M. Grainsize-sensitive viscoelastic relaxation in olivine: Towards a robust laboratory-based model for seismological application. *Phys. Earth Planet. In.*, 183:151–163, 2010. doi: 10.1016/j.pepi.2010.09.005.

13. Katz, R. F., Spiegelman, M., and Langmuir, C. H. A new parameterization of hydrous mantle melting. *Geochem. Geophys. Geosyst.*, 4:1073, 2003. doi: 10.1029/2002GC000433.

14. Namur, O., Collinet, M., Charlier, B., Grove, T. L., Holtz, F., and McCammon, C. Melting processes and mantle sources of lavas on Mercury. *Earth Planet. Sc. Lett.*, 439:117–128, 2016. doi: 10.1016/j.epsl.2016.01.030.

15. Byrne, P. K., Ostrach, L. R., Fassett, C. I., Chapman, C. R., Denevi, B. W., Evans, A. J., Klimczak, C., Banks, M. E., Head, J. W., and Solomon, S. C. Widespread effusive volcanism on Mercury likely ended by about 3.5 Ga. *Geophys. Res. Lett.*, 43:7408–7416, 2016. doi: 10.1002/2016GL069412.
